# Composition and content of phenolic acids and flavonoids among the different varieties, development stages, and tissues of Chinese Jujube (*Ziziphus jujuba* Mill.)

**DOI:** 10.1371/journal.pone.0254058

**Published:** 2021-10-14

**Authors:** Xiaofang Xue, Ailing Zhao, Yongkang Wang, Haiyan Ren, Junjie Du, Dengke Li, Yi Li

**Affiliations:** 1 College of Horticulture, Shanxi Agricultural University, Taigu, Shanxi, People’s Republic of China; 2 Research Institute of Pomology, Shanxi Agricultural University, Shanxi Key Laboratory of Germplasm Improvement and Utilization in Pomology, Taiyuan, Shanxi, People’s Republic of China; Bangabandhu Sheikh Mujibur Rahman Agricultural University, BANGLADESH

## Abstract

The composition and content of phenolic acids and flavonoids among the different varieties, development stages, and tissues of Chinese jujube (*Ziziphus jujuba* Mill.) were systematically examined using ultra-high-performance liquid chromatography to provide a reference for the evaluation and selection of high-value resources. Five key results were identified: (1) Overall, 13 different phenolic acids and flavonoids were detected from among the 20 excellent jujube varieties tested, of which 12 were from the fruits, 11 from the leaves, and 10 from the stems. Seven phenolic acids and flavonoids, including (+)-catechin, rutin, quercetin, luteolin, spinosin, gallic acid, and chlorogenic acid, were detected in all tissues. (2) The total and individual phenolic acids and flavonoids contents significantly decreased during fruit development in *Ziziphus jujuba cv*.Hupingzao. (3) The total phenolic acids and flavonoids content was the highest in the leaves of *Ziziphus jujuba cv*.Hupingzao, followed by the stems and fruits with significant differences among the content of these tissues. The main composition of the tissues also differed, with quercetin and rutin present in the leaves; (+)-catechin and rutin in the stems; and (+)-catechin, epicatechin, and rutin in the fruits. (4) The total content of phenolic acid and flavonoid ranged from 359.38 to 1041.33 μg/g FW across all examined varieties, with *Ziziphus jujuba cv*.Jishanbanzao having the highest content, and (+)-catechin as the main composition in all 20 varieties, followed by epicatechin, rutin, and quercetin. (5) Principal component analysis showed that (+)-catechin, epicatechin, gallic acid, and rutin contributed to the first two principal components for each variety. Together, these findings will assist with varietal selection when developing phenolic acids and f lavonoids functional products.

## Introduction

Jujube (*Ziziphus jujuba* Mill.), originated in China [1], has been cultivated for more than 7000 years and has so rich germplasm resources are available. As the main type of dried fruit produced in China, jujube is an economically important forest tree and was known as one of the “five fruits” in ancient times, along with peach [*Prunus persica* (L.) Batsch], plum (*P*. *domestica* L.), apricot (*P*. *armeniaca*L.), and chestnut (*Castanea* spp.) [2, 3]. The fruit of jujube contains general nutrients and a variety of functional components, including sugars [4, 5], organic acids [6], vitamins C [7], flavonoids [8], cyclic adenosine monophosphate (cAMP), cyclic guanosine monophosphate (cGMP) [9], alkaloid [10], etc., making it become a medicine and food homologous product. Phenolic acids and flavonoids are important secondary metabolites with many biological uses, including antioxidants, antiaging, sedation and hypnosis, in lowering blood fat and pressure, as well as protective functions in the liver and cardiovascular, and cerebrovascular systems [11, 12]. Phenolic acids and flavonoids are among the most important functional nutrients in jujube, and are found in the fruit [13–15], buds [16], leaf [17, 18], and kernels [19]. Therefore, analyzing the phenolic acids and flavonoids composition and content among different jujube varieties and tissues is important for the efficient utilization of jujube germplasm resources.

Plant tissues are the sources of phenolics and flavonoids [20–23]. Phenolic acids in plant include different hydroxybenzoic acids [24] and hydroxycinnamic acids [25] and flavonoids includes flavonols, flavones, flavanols, flavanones, etc. [26, 27]. Several recent studies have investigated jujube flavonoids. Zhou et al. [28] showed that the average total flavonoid content of the fruits of 10 jujube varieties ranged from 1.36 to 5.63 mg/g, whereas Kou et al. [29] found significant differences in the total flavonoid content and antioxidant activity among 15 jujube varieties. In addition, using high-performance liquid chromatography (HPLC), Geng et al. [30] showed that *Ziziphus jujuba cv*. Shanbeiyuanhongzao had the highest rutin content (288.21 μg/g) among eight jujube varieties. Li et al. [31] discovered that the leaves flavonoid content and composition significantly differs among different varieties. Nonetheless, only few studies have been conducted on jujube phenolic acids.

Previous studies on jujube flavonoids have mainly focused on extraction methods [32], determination of the total content, and the content of a few monomer compositions using a narrow range of materials and tissues. Consequently, there is a lack of systematic research on phenolic acids and flavonoids compositions and contents in different varieties, development stages, and tissues. Therefore, in the present study, this variation among 20 representative jujube varieties from the main jujube producing areas in China were analyzed to provide a reference point for further research and for the utilization of jujube phenolic acids and flavonoids.

## Materials and methods

### Plant materials

**Fig 1:**
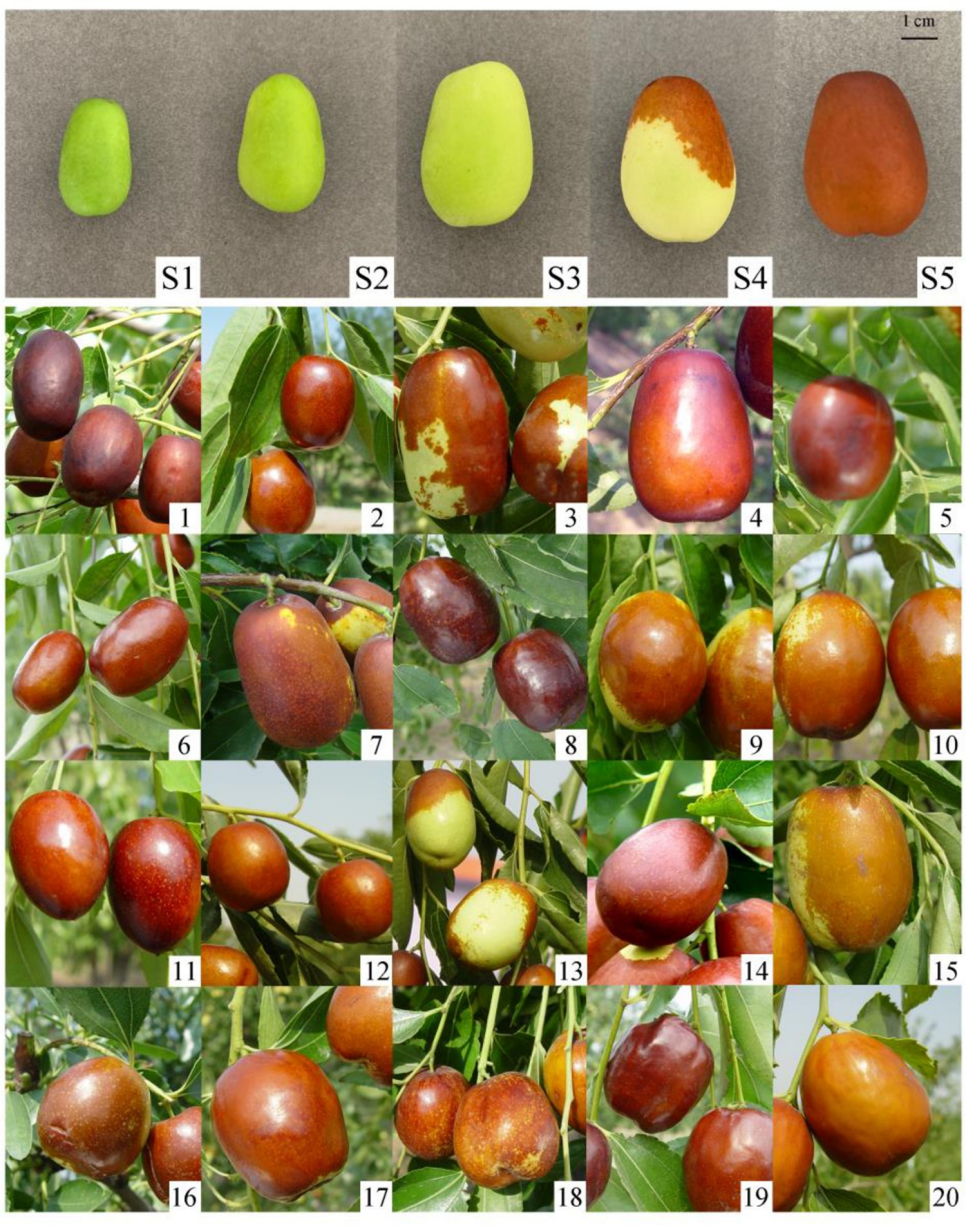
Different development stages of the *Ziziphus jujuba* cv. Hupingzao and red stage fruits from the 20 varieties.

This experiment was performed at the Shanxi Key Laboratory of Germplasm Improvement and Utilization in Pomology of the Research Institute of Pomology, Shanxi Academy of Agricultural Sciences, China. In all, 20 excellent jujube varieties from the main jujube producing areas in China were selected as test materials (Fig 1, S1 Table), all of which were collected from the National Jujube Germplasm Repository. The sample trees were 30-year-old fruit-bearing trees grown in 2.5 m × 3.0 m spaces and subjected to the same cultivation and management practices. Nine sample trees of each variety, with three biological replicates of three trees each were selected.

Fruit samples were collected from 2~5-year-old sections of the trees at the full red mature stage. All sample fruits were hanged outside the tree canopies, the same size and maturity, free from diseases and insect pests. Collection was according to the standard method for jujube [33]. In addition, fruit samples of *Ziziphus jujuba cv*.Hupingzaowere collected at five development stages: young fruit (S1; 40 days after the full-blossom period), expanding fruit (S2; 65 days after the full-blossom period), white maturity (S3; when the surface of the fruit had faded from green to white), half-red maturity (S4), and full red maturity (S5) (Fig 1). Mature leaves and stems from the middle of hanging branches and bearing shoots on 2–5-year-old sections of *Ziziphus jujuba cv*.Hupingzao trees were also collected at the white maturity stage (S3). Each collected sample was washed with tap water and then with distilled water, following which the surface was dried and the sample was placed in a bag. The bag was immediately placed in a freezer at −80°C for later use.

### Reagents

A total of 14 phenolic acids and flavonoids standards with a purity more than 98% were purchased from Sigma. These included (+)-catechin, epicatechin, gallic acid, ferulic acid, chlorogenic acid, caffeic acid, rutin, spinosin, quercetin, phloridzin, isorhamnetin, luteolin, kaempferol, and jujubosideA. In addition, chromatographic pure methanol, formic acid, and acetonitrile were purchased from Merck.

### Instruments

Ultra HPLC (UPLC) was performed using Acquity UPLC H-Class system (Waters), Acquity UPLC UV detector, and Acquity UPLC HSS T3 column (2.1 mm × 100 mm, 1.8 μm) (Waters). Other equipments included an Oasis HLB solid-phase extraction (SPE) column (6 cc/200 mg) (Waters), a 0.22 μm microporous membrane (Tianjin Jinteng Company, China), analytical balance (Sartorius BS BP), centrifuge (SIGMA 3-18K), and SB-5200 DTDN Ultrasonic cleaner (Ningbo Xinzhi Biotechnology Co. Ltd., China).

### Sample extraction

Sample pretreatment was performed as per the methods of Li et al. [34] and Li et al. [35] with some modification. First, 1.0000 ± 0.0005 g of each prepared sample was weighed into a 50 mL centrifuge tube. Then, 10 mL extract (methanol:water:formic acid = 70:29:1) was added and the mixture was treated ultrasonically for 30 min at 50°C and then centrifuged for 10 min at 10,000 rpm, following which the residue was extracted once again. The two supernatants were combined and poured into a 25 mL brown volumetric flask, and the volume was brought to 25 mL by adding the extract. This sample was then passed through an SPE column and filtered through a 0.22 μm membrane before use.

### Chromatographic conditions

UPLC was performed using Acquity UPLC HSS T3 column (2.1 mm × 100 mm, 1.8 μm) with methanol as mobile phase A, and 0.2% formic acid aqueous solution as mobile phase B, and using the gradient method for elution (Table 1). The column temperature was maintained at 35°C, the injected volume was 3 μL, and the flow rate was 0.25 mL/min. UPLC ultraviolet detector was used for detection using wavelengths of 283 nm and 367 nm and an automatic sampler.

**Table 1:** Mobile phase gradient used in ultra-high performance liquid chromatography.

Time (min)	0.0	1.5	4.5	6.0	10.0	13.0
Flow rate (mL/min)	0.25	0.25	0.25	0.25	0.25	0.25
Methanol (A) (%)	32	32	50	45	70	32
0.2% methanoic acid (B) (%)	68	68	50	55	30	68
Curve	6	3	5	7	6	1

### Standard solution preparation

A 10.0 mg aliquot of each standard was weighed using an electronic analytical balance, dissolved in methanol, stabilized in a 10mL volumetric flask, and then stored at 4°C for later use. In addition, the standard mother liquors were diluted to different concentrations to produce standard curves. Detection was performed using the same chromatographic conditions as described above.

### Statistical analysis

All statistical analyses were conducted using Excel 2007; all assays were performed in triplicates, and all the data are expressed as mean values ± standard deviation. Statistically significant differences were determined using one-way repeated measures analysis of variance performed using SPSS 18.0. Correlation and principal components analyses among the mean values were conducted using Statistical Analysis System v. 9.2 software.

## Results

### Phenolic acids and flavonoids composition of the samples

**Fig 2:**
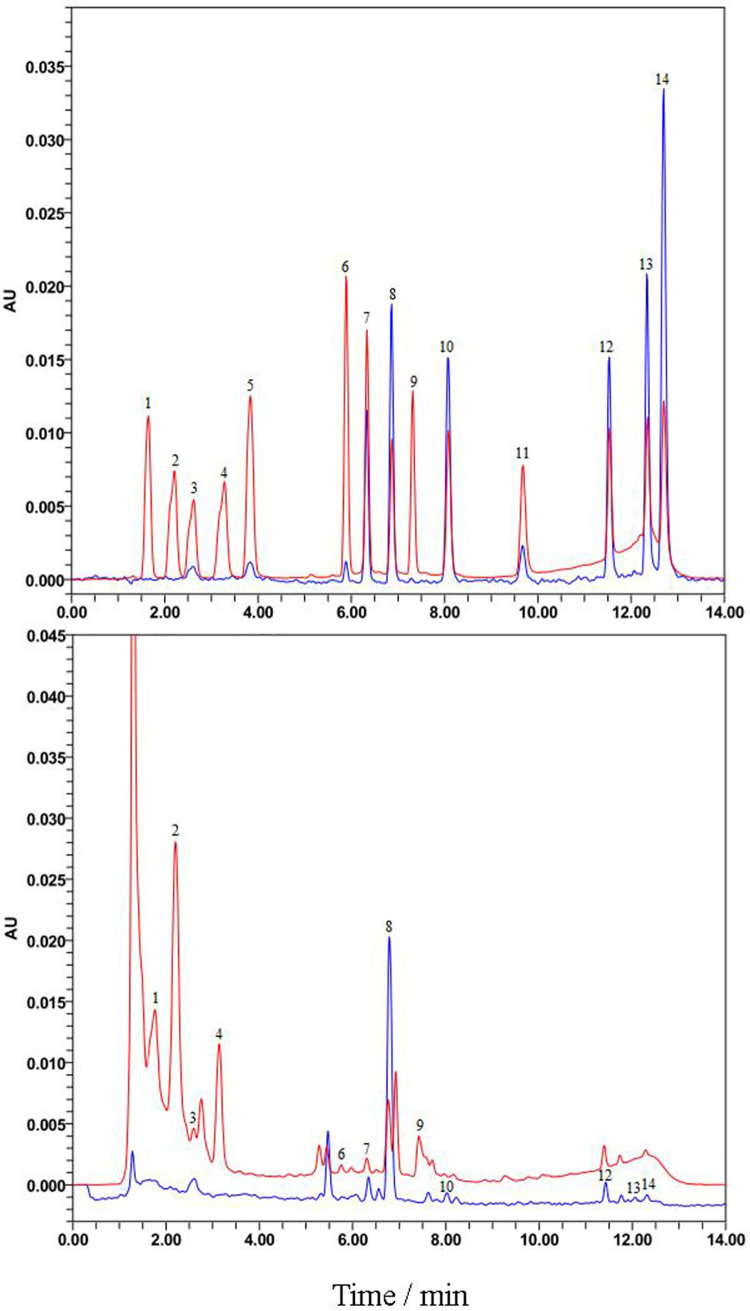
Chromatograms of phenolic acids and flavonoids standards and sample using ultra-high performance liquid chromatography. (A) Standard and (B) sample; red and blue colors represent the test results at 283 nm and 367 nm, respectively. 1. gallic acid; 2. (+)-catechin; 3. chlorogenic acid; 4. epicatechin; 5. caffeic acid; 6. ferulic acid; 7. spinosin; 8. rutin; 9. phloridzin; 10. quercetin; 11. jujubosideA; 12. luteolin; 13. kaempferol; 14.isorhamnetin.

Chromatograms of the 14 phenolic acids and flavonoids standards under optimized analytical conditions are presented in Fig 2A. In total, 13 of these standards, including (+)-catechin, epicatechin, rutin, quercetin, kaempferol, isorhamnetin, spinosin, phloridzin, luteolin, gallic acid, chlorogenic acid, ferulic acid, and caffeic acid, were detected from different varieties, development stages, and tissues, with different combinations in each variety. Of the 14 phenolic acids and flavonoids, 12 were detected in the fruits (Fig 2B), 11 in the leaves, and 10 in the stems. Seven phenolic acids and flavonoids, (+)-catechin, rutin, quercetin, spinosin, luteolin, gallic acid, and chlorogenic acid, were common for all tissues.

### Changes in phenolic acids and flavonoids composition and content among fruit at different development stages

**Fig 3:**
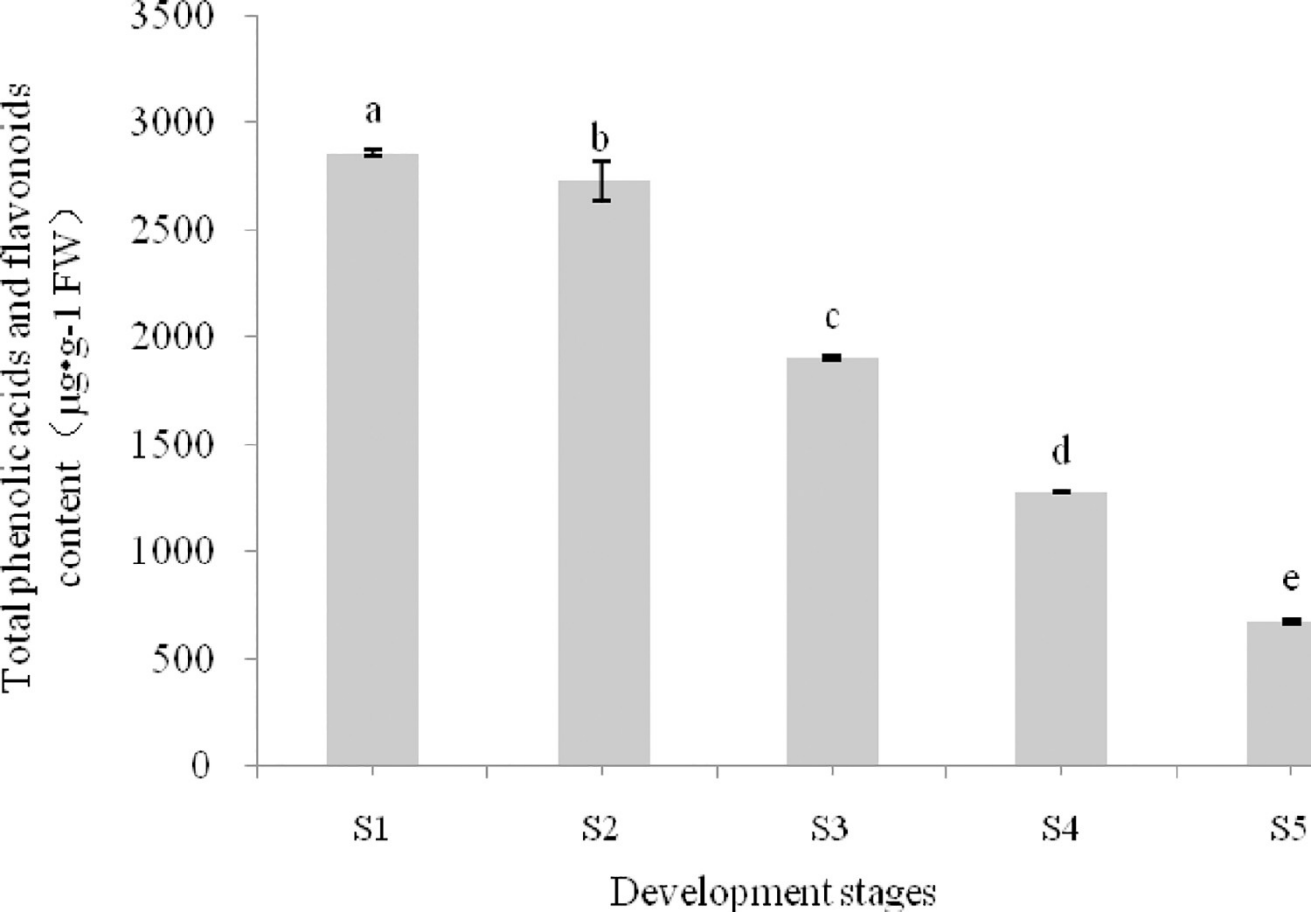
Changes in the total phenolic acids and flavonoids content of the fruit of Chinese jujube (*Ziziphus jujuba*cv. Hupingzao) during development. Bars with different letters are significantly different (Duncan’s test, *P* < 0.05).

The total phenolic acids and flavonoids content of *Ziziphus jujuba* cv. Hupingzao fruit significantly differed depending on the stage of fruit development and decreased as development progressed (Fig 3). There was only a slight decrease from S1 to S2, but this was followed by a rapid decrease from S2 onward. Thus, the phenolic acid and flavonoid content was the highest in S1 (2856.57 μg/g FW) and lowest in S5 (675.34 μg/g FW), showing a difference of 2181.23 μg/g FW.

Ten phenolic acids and flavonoids, (+)-catechin, epicatechin, rutin, quercetin, kaempferol, spinosin, phloridzin, luteolin, gallic acid, and chlorogenic acid, were identified in stages S1–S4, whereas isorhamnetin (1.901 μg/g FW) was also detected in S5; The contents of (+)-catechin, rutin, quercetin, spinosin, and luteolin decreased with fruit development (Table 2). In contrast, the contents of epicatechin, kaempferol, gallic acid, and chlorogenic acid initially increased and then decreased, with the highest content observed in S2, whereas the content of phloridzin peaked in S3.

**Table 2:** Dynamic changes in the phenolic acids and flavonoids composition of Chinese jujube fruits at different development stages.

	**(+)-Catechin**	**Epicatechin**	**Gallic acid**	**Rutin**	**Quercetin**
S1	1364.80±12.38a	692.26±15.32c	370.51±1.07b	283.75±3.28a	54.63±0.54a
S2	908.31±30.99b	1166.14±48.68a	428.40±4.75a	131.91±5.65b	28.68±3.27b
S3	397.31±13.86c	1098.58±2.16b	286.72±9.23c	63.57±0.10d	7.47±0.68d
S4	312.32±1.81d	680.75±6.64c	159.88±3.62d	69.88±2.10c	11.35±0.44c
S5	382.68±5.05c	169.27±6.09d	24.89±1.25e	41.72±0.89e	10.01±1.03c
	**Spinosin**	**Luteolin**	**Kaempferol**	**Chlorogenic acid**	**Phloridzin**
S1	39.73±0.71a	6.06±0.25a	1.05±0.02c	25.78±0.96b	18.00±0.05c
S2	13.29±0.76b	5.15±0.34b	1.55±0.04a	29.28±1.94a	15.43±0.68d
S3	8.32±0.24c	3.68±0.31c	1.50±0.08a	11.64±0.60d	23.05±0.07a
S4	7.42±0.66d	3.77±0.44c	1.49±0.03a	11.31±0.38d	15.18±0.58d
S5	5.15±0.53d	2.95±0.07d	1.19±0.04b	13.69±0.81c	21.90±0.33b

The main phenolic acids and flavonoids in the S1 stage was (+)-catechin (47.78% of the total phenolic acids and flavonoids content), followed by epicatechin (24.23%), gallic acid (12.97%), and rutin (9.93%). In contrast, low levels of quercetin, spinosin, luteolin, kaempferol, chlorogenic acid, and phloridzin were detected. Conversely, the main phenolic acid and flavonoid in the S2, S3, and S4 stages was epicatechin (42.74%, 57.76%, and 53.46%, respectively), followed by (+)-catechin (33.29%, 20.89%, and 24.53%, respectively), gallic acid (15.70%, 15.08%, and 12.55%, respectively), and rutin (4.84%, 3.34%, and 5.49%, respectively). Finally, the main phenolic acid and flavonoid in the S5 stage was (+)-catechin (56.67%), followed by epicatechin (25.06%) and rutin (6.18%), with lower levels of all other compositions. Throughout all stages of fruit development, kaempferol was present at the lowest content, accounting for only 0.04%–0.13% of the total content of phenolic acids and flavonoids.

### Phenolic acids and flavonoids composition and content in different tissues

There were significant differences in the total phenolic acids and flavonoids contents of the fruit, leaves, and stems of *Ziziphus jujuba cv*. Hupingzao at the S3 stage, with the leaves and stems having 5.39 and 3.63 times the phenolic acids and flavonoids content of the fruit, respectively (Table 3). The types of phenolic acids and flavonoids present also differed among the tissues, with isorhamnetin, ferulic acid, and caffeic acid not being detected in the fruit; epicatechin and caffeic acid not being detected in the leaves; and isorhamnetin, kaempferol, and phloridzin not being detected in the stems. In addition, there were significant differences in the main phenolic acids and flavonoids compositions among three tissues. In the fruit, the content of epicatechin was the highest (57.76% of the total content), followed by (+)-catechin (20.89%), gallic acid (15.08%), and rutin (3.34%). In the leaves, the content of quercetin and rutin was the highest (41.43% and 39.94%, respectively), followed by chlorogenic acid (6.43%) and gallic acid (5.74%). In the stems, the content of (+)-catechin was the highest (54.78%), followed by rutin (30.51%).

**Table 3:** Composition and content of phenolic acids and flavonoids among different tissues of Chinese jujube (*Ziziphus jujuba* cv. Hupingzao).

**Tissue**	**(+)-Catechin**	**Epicatechin**	**Rutin**	**Quercetin**	**Isorhamnetin**	**Kaempferol**	**Spinosin**
Fruit	397.31±13.86b	1098.58±2.16a	63.57±0.10c	7.47±0.68c	ND	1.50±0.08b	8.32±0.24c
Leaves	218.71±31.21c	ND	4095.94±42.23a	4247.95±101.92a	47.76±1.71	31.56±1.77a	207.66±9.45a
stems	3776.86±58.15a	261.52±15.70b	2103.46±52.08b	115.10±17.61b	ND	ND	76.59±5.98b
**Tissue**	**Phloridzin**	**Luteolin**	**Gallic acid**	**Ferulic acid**	**Chlorogenic acid**	**Caffeic acid**	**Total**
Fruit	23.05±0.07b	3.68±0.31c	286.72±9.23b	ND	11.64±0.60c	ND	1901.83±6.45c
Leaves	80.36±5.34a	48.99±7.75a	588.26±6.42a	28.28±0.71a	658.97±21.50a	ND	10,254.4±99.91a
stems	ND	34.99±0.68b	155.30±20.05c	20.45±0.75b	251.40±14.55b	98.73±0.32	6894.39±96.50b

All values are in μg/g FW.

Note: ND, represents not detected. Values followed by different letters are significantly different (Duncan’s test, *P* < 0.05).

### Phenolic acids and flavonoids compositions and contents among the fruits of different varieties

The phenolic acids and flavonoids composition and content of the fruits of 20 representative jujube varieties at the full red stage (S5) are presented in Table 4. The total contents varied significantly among the varieties examined, ranging from 359.382 to 1041.333 μg/g FW (average value, 605.490 μg/g FW), representing a 2.90 fold difference. Among the varieties, *Ziziphus jujuba cv*.Jishanbanzao had the highest content, followed by *Ziziphus jujuba cv*.Zaoqiangpozao, *Ziziphus jujuba cv*.Yongjihamazao, *Ziziphus jujuba cv*.Jiaochengjunzao, and *Ziziphus jujuba cv*.Yunchengxiangzao.

**Table 4:** Compositions and contents of phenolic acids and flavonoids in full red stage fruits of 20 Chinese jujube (*Ziziphus jujuba*) varieties.

No.	(+)-Catechin	Epicatechin	Rutin	Quercetin	Isorhamnetin	Kaempferol	Spinosin	Phloridzin	Luteolin	Gallic acid	Ferulic acid	Chlorogenic acid	Total contents
1	620.53±1.19a	231.34±4.14c	72.28±0.66f	7.89±0.58efg	1.55±0.04ef	1.45±0.03c	6.00±0.47cd	6.67±0.48j	2.41±0.08j	69.88±2.09c	ND	21.35±0.86d	1041.33±5.18a
2	367.78±8.93f	285.07±14.90a	92.41±3.36c	14.36±0.78c	ND	1.04±0.04fg	7.73±0.21ab	7.12±0.17ij	2.78±0.15gh	142.91±0.72a	1.03±0.03e	24.66±1.21bc	946.88±21.41b
3	294.67±5.82h	261.84±4.54b	86.65±3.35d	29.07±2.39a	2.26±0.10a	1.63±0.03b	8.23±0.43a	4.03±0.15l	2.71±0.22ghi	78.80±4.89b	ND	30.64±0.58a	800.52±11.22c
4	632.39±9.02a	40.34±1.65k	31.13±0.60n	7.74±0.86efg	ND	1.31±0.01d	6.09±0.79c	26.98±0.16b	3.59±0.08c	6.15±0.51m	ND	16.60±0.65fg	772.31±10.10d
5	591.38±8.28b	95.21±5.58h	28.86±0.52n	ND	2.13±0.07b	1.69±0.04ab	ND	1.22±0.10o	2.87±0.17fg	10.71±0.23l	ND	21.44±2.26d	755.51±9.47de
6	464.78±19.54d	80.08±4.37ij	108.94±3.00a	8.64±0.13de	ND	1.46±0.08c	4.87±0.53ef	5.22±0.32k	4.33±0.17b	37.64±2.12i	ND	23.87±1.87c	739.81±22.30e
7	382.68±5.05e	169.27±6.09e	41.72±0.89 l	10.01±1.03d	1.90±0.01c	1.19±0.04e	5.15±0.53def	21.90±0.33d	2.95±0.07fg	24.89±1.25j	ND	13.69±0.81h	675.34±10.89f
8	543.02±10.80c	33.96±6.35k	37.70±0.23m	4.16±0.29j	ND	1.20±0.03e	5.19±0.76def	3.08±0.04m	3.13±0.02ef	9.37±0.33lm	ND	29.37±2.42a	670.19±4.64f
9	246.48±5.55j	155.68±4.84f	76.42±0.37e	6.79±0.39fgh	1.53±0.02ef	1.42±0.03c	7.12±0.86b	4.77±0.13kl	2.54±0.04hij	56.56±1.61e	1.34±0.03b	18.54±0.85ef	579.18±10.09g
10	196.50±5.94lm	182.07±6.28d	42.50±0.81kl	6.24±0.94ghi	ND	1.25±0.05de	3.26±0.17h	23.90±0.65c	3.55±0.22c	51.60±0.45f	ND	18.35±0.80ef	529.21±4.86h
11	276.26±9.32i	82.91±7.61i	60.00±0.64h	21.03±2.56b	ND	0.97±0.02g	5.39±0.27cde	7.64±1.08i	3.75±0.14c	21.51±0.72k	ND	26.15±0.99bc	505.60±19.52i
12	159.08±3.03n	144.81±1.11g	98.82±1.04b	5.72±0.48hij	ND	1.42±0.04c	8.34±0.41a	2.21±0.12n	2.50±0.09ij	50.60±3.17fg	ND	25.08±0.38bc	498.56±1.10i
13	353.03±6.60g	35.67±4.32k	38.67±2.04m	8.54±0.35def	1.48±0.03ef	1.19±0.07e	3.15±0.13h	17.89±0.08e	2.35±0.09j	8.16±0.26lm	ND	21.71±1.93d	491.84±11.08i
14	199.53±9.90lm	98.32±3.23h	67.28±2.17g	14.13±0.98c	1.90±0.05c	1.74±0.03a	5.26±0.75cdef	10.58±0.39h	3.24±0.26de	65.20±2.98d	ND	24.56±1.44bc	491.73±14.49i
15	264.38±8.84i	32.44±1.43kl	48.24±0.67j	6.69±0.85ghi	1.73±0.09d	1.46±0.08c	4.80±0.33ef	29.00±1.13a	4.62±0.25a	21.37±0.82k	1.29±0.06b	17.92±0.51ef	433.93±7.83j
16	193.90±14.98m	77.89±2.25ij	57.34±0.87i	12.75±0.45c	1.49±0.03ef	1.18±0.08e	4.39±0.41fg	6.80±0.37ij	3.59±0.11c	49.36±2.32fg	1.19±0.04cd	20.08±1.58de	429.94±14.24j
17	210.68±4.35l	72.41±3.97j	42.57±1.43kl	4.91±0.16ij	1.47±0.03f	1.18±0.03e	5.14±0.41def	21.62±0.58d	3.48±0.31cd	42.58±1.51h	ND	15.11±0.28gh	421.15±2.78jk
18	194.01±6.21m	70.54±2.55j	45.23±0.94k	4.31±0.16j	1.57±0.03e	1.18±0.11e	4.92±0.43ef	16.96±0.28f	2.46±0.05ij	47.63±0.64g	1.11±0.02de	15.33±0.39gh	405.24±5.01k
19	228.41±7.42k	24.20±3.54lm	42.30±1.23l	12.83±0.63c	1.85±0.04c	1.07±0.05f	3.84±0.13gh	4.15±0.28l	4.53±0.07ab	19.99±1.39k	2.53±0.1a	26.71±0.75b	372.41±6.08l
20	242.61±3.91jk	16.51±1.56m	43.37±0.67kl	4.04±0.65j	ND	1.17±0.03e	3.84±0.19gh	14.83±0.80g	1.99±0.03k	8.16±0.29lm	1.27±0.03bc	21.60±1.58d	359.38±1.77l

All values are in μg/g FW.

Note: ND, not detected. values followed by different letters are significantly different (Duncan’s test, *P* < 0.05).

There were also significant differences in the phenolic acids and flavonoids compositions among the different varieties examined. In total, 12 phenolic acids and flavonoids, including (+)-catechin (average of the total content = 55.95%), epicatechin (18.71%), rutin (9.76%), gallic acid (7.30%), chlorogenic acid (3.58%), phloridzin (2.00%), quercetin (1.65%), spinosin (0.89%), luteolin (0.53%), isorhamnetin (0.29%), ferulic acid (0.23%), and kaempferol (0.22%) were identified in the full red fruits of the 20 jujube varieties. Among these, isorhamnetin and ferulic acid were only detected in some varieties, whereas quercetin and spinosin were present in all varieties except *Ziziphus jujuba cv*.Yunchengxiangzao. In all varieties, the content of (+)-catechin was the highest (31.91%–81.03%), followed by epicatechin, rutin, gallic acid, and chlorogenic acid; the content of other flavonoids, such as quercetin, spinosin, phloridzin, luteolin, and kaempferol were low. The varieties with high total phenolic acids and flavonoids content did not necessarily have a high content of every phenolic acid and flavonoid.

### Correlations among the different phenolic acids and flavonoids compositions

**Fig 4:**
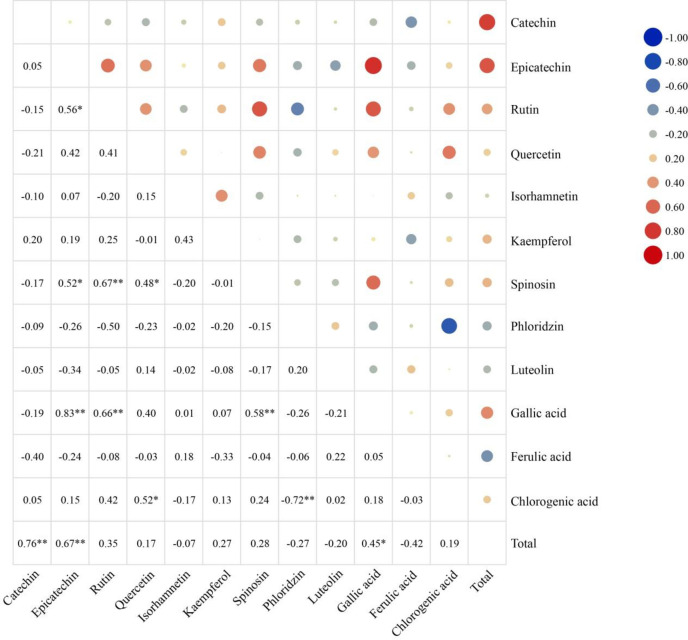
Correlations between the different phenolic acids and flavonoids compositions in the 20 Chinese jujube varieties. * and ** indicate significance at *P* < 0.05 and *P* < 0.01, respectively.

Correlation analysis of the different phenolic acids and flavonoids compositions of the 20 varieties (Fig 4) revealed that (+)-catechin and epicatechin contents were significantly positively correlated with the total content (*P* < 0.01); gallic acid content was significantly positively correlated with the total phenolic acids and flavonoids content (correlation coefficient = 0.45; *P* < 0.05). Epicatechin content was significantly positively correlated with gallic acid (*P* < 0.01) as well as rutin and spinosin (*P* < 0.05); rutin content was significantly positively correlated with gallic acid and spinosin contents (*P* < 0.01); quercetin content was significantly positively correlated with chlorogenic acid and spinosin contents (*P* < 0.01); spinosin content was significantly positively correlated with gallic acid content (*P* < 0.01); and phloridzin content was significantly negatively correlated with chlorogenic acid content (correlation coefficient = -0.72; *P* < 0.01).

### Principal component analysis

Principal component analysis (PCA) identified two components that explained 97.75% of the total variation in the phenolic acids and flavonoids composition of the 20 varieties (Table 5). The first principal component (PC1) contributed 74.60%. Large, and positive values were associated with (+)-catechin, suggesting that it greatly contributed to PC1. The second principal component (PC2) contributed 23.15% of the total variation. Large and positive values were associated with epicatechin, gallic acid, and rutin, suggesting that these greatly contributed to PC2.

**Table 5:** Principal component analysis of phenolic acids and flavonoids compositions in the 20 jujube varieties.

Compositions	Eigenvectors	
	Prin1	Prin2
(+)-Catechin (μg/g FW)	0.998	-0.011
Epicatechin (μg/g FW)	0.030	0.927
Rutin (μg/g FW)	-0.023	0.169
Quercetin (μg/g FW)	-0.009	0.033
Isorhamnetin (μg/g FW)	-0.001	0.001
Kaempferol (μg/g FW)	0.000	0.000
Spinosin (μg/g FW)	-0.002	0.012
Phloridzin (μg/g FW)	-0.005	-0.029
Luteolin (μg/g FW)	-0.000	-0.003
Gallic acid (μg/g FW)	0.040	0.331
Ferulic acid (μg/g FW)	-0.002	-0.002
Chlorogenic acid (μg/g FW)	0.001	0.009
Proportion (%)	74.60	23.15
Cumulative (%)	74.60	97.75

**Fig 5:**
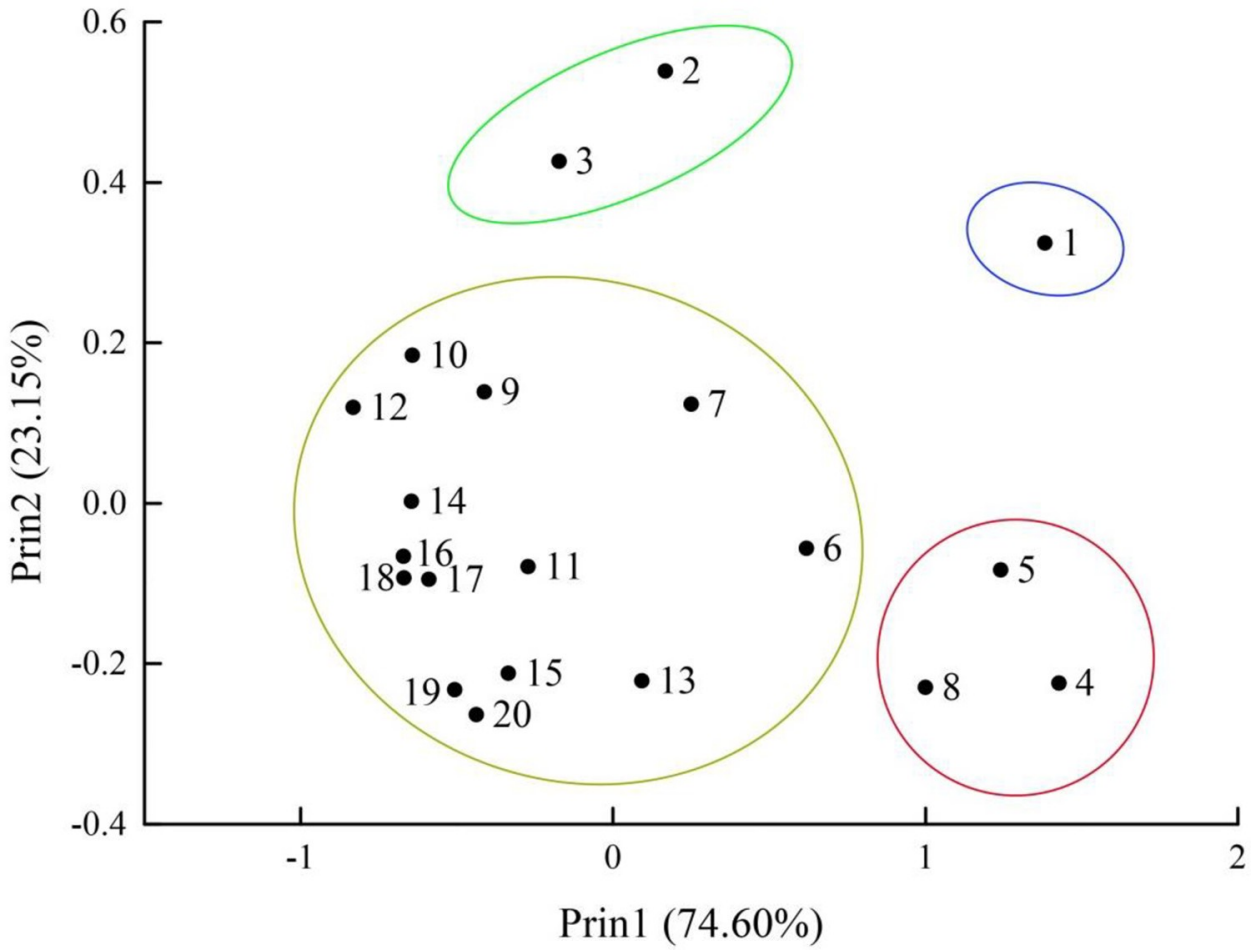
Scatterplot of principal component analysis based on phenolic acids and flavonoids composition of 20 jujube varieties. The four circles indicate the varieties belonging to the top two principal components.

Scatterplots of the PCA based on the phenolic acids and flavonoids compositions in the 20 Chinese jujube varieties showed that *Ziziphus jujuba cv*.Jishanbanzao belonged to the first group (Fig 5) characterized by higher (+)-catechin and epicatechin content. *Ziziphus jujuba cv*.Zaoqiangpozao and *Ziziphus jujuba cv*.Yongjihamazao belonged to the second group characterized by high epicatechin, rutin, and gallic acid levels. *Ziziphus jujuba cv*.Jiaochengjunzao, *Ziziphus jujuba cv*.Yunchengxiangzao, and *Ziziphus jujuba cv*.Yuanlingzao belonged to the third group characterized by high (+)-catechin and low epicatechin, rutin, and gallic acid levels. The remaining varieties belonged to the fourth group characterized by medium composition levels of each composition. These results provide a reference for the selection of high phenolic acids and flavonoids composition and content varieties.

## Discussion

Phenolic acids and flavonoids are some of the most important functional nutrients in jujube. We therefore systematically evaluated the phenolic acids and flavonoids composition and content among different varieties, development stages, and tissues of Chinese jujube. First, we selected *Ziziphus jujuba cv*. Hupingzao as our experimental material to analyze the phenolic acids and flavonoids composition among the different development stages and tissues. *Ziziphus jujuba cv*. Hupingzao, from the main production province of Shanxi, China, is a Chinese jujube variety that has been awarded the “National Geographical Indicated Products”, validating its selection as a representative sample. Second, we selected 20 excellent varieties of Chinese jujube from the main production areas of Shanxi, Shandong, Henan, Hebei, and Shannxi provinces to determine the phenolic acids and flavonoids composition and content of the fruits during their full maturity stage. Fully matured fruits—namely, full red jujube fruits—are extremely nutritious and can replenish qi; while nourishing and soothing the nerves [36]. Consequently, they are often used to enhance the efficacy of medicines and are the main part of the jujube plant that is the most utilized. From a practical application point of view, it is reasonable that the phenolic acids and flavonoids content of the 20 representative Chinese jujube varieties included in this study should be analyzed for fruits at the full red stage rather than at the young fruit period, during which the content is high. We undertook a comprehensive analysis of the possible phenolic acids and flavonoids in jujube by selecting 14 standards on the basis of previously published research. A total of 13 phenolic acids and flavonoids were detected in the jujube samples. An unknown peak was also observed in the test samples, but because that its peak area was small, it was not counted among the main phenolic acids and flavonoids. However, the identity of this unknown substance should be determined in the future using mass spectrometry and other techniques.

We found that the total phenolic acids and flavonoids content of the jujube fruit decreased, as the fruit developed; this is consistent with the results obtained by Zhao et al. [37] and Shen et al. [38] for jujube and Huang et al. [39] for kiwifruit (*Actinidia deliciosa* Planch) determined using spectrophotometry. In addition to phloridzin, the content of other phenolic acids and flavonoids also decreased with fruit development, supporting the results of Xia et al. [40] who studied the dynamic changes in the flavonoids composition in apricot during fruit development. The dynamic change in the content of each phenolic acid and flavonoid with fruit development is due to the variation in the balance between synthesis, transportation, and decomposition. Our results indicated that the main stage of phenolic acids and flavonoids synthesis in the Chinese jujube fruit occurred in the young fruit, and the compositions in the fruit at this stage were actively metabolized. Young naturally shed fruits can be used to extract phenolic acids and flavonoids and other active ingredients to maximize the utilization of resources.

The detected phenolic acids and flavonoids composition varied among the different varieties, development stages, and tissues, with 12 phenolic acids and flavonoids being detected in the fruits, 11 in the leaves, and 10 in the stems. However, seven phenolic acids and flavonoids were common to all samples. Three flavonoids were reported in the leaves of *Amaranthus*.*tricolor* [27, 41]; nine in the drought-tolerant vegetable amaranth leaves [24, 26], salt-tolerant vegetable amaranth leaves [42], and *A*.*gangeticus* leaves [25]; and eight in the leaves of red and green amaranth [43]. Isorhamnetin and ferulic acid were detected only in the fruits of some varieties; therefore, it was speculated that they had varietal specificity. Moreover, epicatechin was not detected in the leaves, isorhamnetin, kaempferol, and phloridzin were not detected in the stems, and caffeic acid was only detected in the stem. It can be inferred that there were differences in the detected phenolic acids and flavonoids among different parts of the plant. In contrast, the leaves and stems had higher total contents than the fruit, and the main phenolic acids and flavonoids also differed among these tissues, with the contents of quercetin, (+)-catechin, and epicatechin being the highest in the leaves, stems, and fruit at the white maturity stage (S3), respectively. The results indicated that the phenolic acids and flavonoids contents varied greatly among different parts. The reasons for these differences in the monomer compositions of the different tissues need to be further studied. Flavonoids in the leaves show a variety of biological properties, such as antioxidant, neuroprotective, and bioprotective properties [44, 45], indicating that it would be beneficial to increase the utilization of the flavonoid-rich leaves and stems to increase the economic benefit of jujube as a forest tree. Zhang et al. [46] previously showed that the sprouting stage of walnut (*Juglans sigillata* Dode) leaves had the highest content of flavonoids. Therefore, it would also be useful to study the dynamic changes in jujube leaves at different development stages to determine when the flavonoid content is the highest.

Many studies have confirmed that the composition and content of flavonoids vary among fruit species. For instance, flavanones such as naringin and hesperidin are the main flavonoids in citrus [47, 48], whereas the flavonoid dihydrochalcone is found in apples [49]. Furthermore, the flavonoid composition and content also vary among different types and varieties of the same fruit species. For example, different types of peaches have been shown to have different flavonoid compositions, with epicatechin being the main composition in juicy peach and peach and catechin being the main composition in nectarine [50]. In the present study, we found that the main phenolic acids and flavonoids in the fully mature Chinese jujube fruit were the flavonoids (+)-catechin, epicatechin, and rutin; and the phenolic acids gallic acid; of these, the content of(+)-catechin was the highest. The bioactive functions of specific phenolic acids and flavonoids depend on their composition. For example, catechins can inhibit breast, liver, colorectal, and other types of cancers [51]; rutin functions as an antibacterial and anti-inflammatory [52, 53] substance; gallic acid shows antioxidant, anti-inflammatory, and nephroprotective properties [54]. In this study, we identified the differences between the flavonoids of Chinese jujube fruit and other fruit trees, and analyzed the main phenolic acids and flavonoids in Chinese jujube fruits. These results will provide a reference for the efficient utilization of jujube germplasm resources and deeper research and development of relevant functional products.

Through the multivariate statistical method of PCA, we selected a small number of important variables through linear transformation, that could reflect the information of the original variables as thoroughly as possible; this method is used for dimension reduction in mathematics. In this study, we identified two components using PCA, including four indices—of (+)-catechin, epicatechin, gallic acid, and rutin. Based on these indices, we grouped the 20 different varieties into four categories with distinct characteristics. Of them, *Ziziphus jujuba cv*. Jishanbanzao, *Ziziphus jujuba cv*. Jiaochengjunzao, *Ziziphus jujuba cv*. Yunchengxiangzao, and *Ziziphus jujuba cv*. Yuanlingzao are rich in (+)-catechin; *Ziziphus jujuba cv*. Zaoqiangpozao and *Ziziphus jujuba cv*. Yongjihamazao are rich in epicatechin, gallic acid, and rutin. These varieties could therefore be used as raw materials to develop of functional products or as breeding materials for new varieties with correspondingly high compositions. In the future, it would be useful to investigate the biological functions as well as the metabolic mechanism of the different phenolic acids and flavonoids using molecular biological methods. Moreover, our study found no correlation between the origin of the varieties and PCA results. Although the varieties included in this study originated from different ecological conditions, the samples were collected from the same ecological conditions, and each variety had been introduced to the conservation place since several decades, which may be the reason for this result. This provides us with new avenues of research, to explore the differences in the phenolic acids and flavonoids composition and content of these important varieties under different ecological conditions, to further clarify the impact of ecological conditions on the contents of phenolic acids and flavonoids and other bioactive substances.

## Conclusions

We explored the composition and content of phenolic acids and flavonoids among different varieties, development stages, and tissues of Chinese jujube. A total of 13 phenolic acids and flavonoids were detected among the different samples, including (+)-catechin, epicatechin, rutin, quercetin, kaempferol, isorhamnetin, spinosin, phloridzin, luteolin, gallic acid, chlorogenic acid, ferulic acid, and caffeic acid. The total content and composition of phenolic acids and flavonoids decreased with development of the fruit. In addition, the total content varied among different tissues, with the leaves having the highest content and the fruit having the lowest. The main phenolic acids and flavonoids in each tissue also differed, with the highest content of(+)-catechin, epicatechin, and rutin in the fruit; quercetin and rurin in the leaves; and (+)-catechin and rutin in the stem. Finally, (+)-catechin was identified as the main composition in the 20 varieties examined, but there were significant differences in the total contents. Using PCA, we grouped the 20 varieties and screened them with higher contents of important compositions. In future studies, we should purposefully develop and utilize different varieties and tissues according to their individual composition and content.

## Supporting information

S1 Table Details of the 20 Chinese jujube varieties. (DOCX)
