# Supplementary material for: Composition and content of phenolic acids and flavonoids among the different varieties, development stages, and tissues of Chinese Jujube (Ziziphus jujuba Mill.)
Source: PLoS One. 2021 Oct 14;16(10):e0254058. doi: 10.1371/journal.pone.0254058 (PMC8516285; doi:10.1371/journal.pone.0254058)
Supplement: S1 Table — (DOCX) [file pone.0254058.s001.docx]

**S1 Table. Details of the 20 Chinese jujube varieties**

| **No.** | **Name** | **Origin** | **Collected year** | **Fruit color** |
| --- | --- | --- | --- | --- |
| 1 | Jishanbanzao | Jishan, Shanxi, China | 1983 | Red purple |
| 2 | Zaoqiangpozao | Zaoqiang, Hebei, China | 1983 | Red purple |
| 3 | Yongjihamazao | Yongji, Shanxi, China | 1981 | Red |
| 4 | Jiaochengjunzao | Jiaocheng, Shanxi, China | 1981 | Red purple |
| 5 | Yunchengxiangzao | Salt lake district, Shanxi, China | 1981 | Red purple |
| 6 | Guantanzao | Xiangfen, Shanxi, China | 1981 | Red purple |
| 7 | Hupingzao | Taigu, Shanxi, China | 1981 | Red purple |
| 8 | Yuanlingzao | Ciping, Shandong, China | 1983 | Red purple |
| 9 | Lengbaiyuzao | Taigu, Shanxi, China | 1983 | Red |
| 10 | Beijingjidanzao | Haidian district, Beijing, China | 1985 | Red |
| 11 | Xinzhenghuizao | Xinzheng, Henan, China | 1983 | Red purple |
| 12 | Dalifengmiguan | Dali, Shaanxi, China | 1983 | Red |
| 13 | Cangxianjinsixiaozao | Cangxian, Hebei, China | 1983 | Red |
| 14 | Zanhuangdazao | Zanhuang, Hebei, China | 1983 | Red purple |
| 15 | Binxianjinzao | Binxian, Shaanxi, China | 1983 | Red |
| 16 | Pingguozao | Daming, Hebei, China | 1983 | Red |
| 17 | Linyilizao | Linyi, Shanxi, China | 1983 | Red |
| 18 | Xiajindabailing | Linqing, Shanxi, China | 1985 | Red |
| 19 | Puyanghetaowen | Puyang, Henan, China | 1985 | Red purple |
| 20 | Shandonglizao | Qingyun, Shandong, China | 1985 | Red |
